# Coral reef fish perceive lightness illusions

**DOI:** 10.1038/srep35335

**Published:** 2016-10-17

**Authors:** Elisha E. Simpson, N. Justin Marshall, Karen L. Cheney

**Affiliations:** 1 School of Biological Sciences, The University of Queensland, Brisbane, Queensland, 4072, Australia; 2 Queensland Brain Institute, The University of Queensland, Brisbane, Queensland, 4072, Australia

## Abstract

Visual illusions occur when information from images are perceived differently from the actual physical properties of the stimulus in terms of brightness, size, colour and/or motion. Illusions are therefore important tools for sensory perception research and from an ecological perspective, relevant for visually guided animals viewing signals in heterogeneous environments. Here, we tested whether fish perceived a lightness cube illusion in which identical coloured targets appear (for humans) to return different spectral outputs depending on the apparent amount of illumination they are perceived to be under. Triggerfish (*Rhinecanthus aculeatus)* were trained to peck at coloured targets to receive food rewards, and were shown to experience similar shifts in colour perception when targets were placed in illusory shadows. Fish therefore appear to experience similar simultaneous contrast mechanisms to humans, even when targets are embedded in complex, scene-type illusions. Studies such as these help unlock the fundamental principles of visual system mechanisms.

Visual perception is influenced by factors other than information provided by the retinal image: higher-level processes adjust visual information to account for variation in visual conditions, such as shadows, filters, or haze. Illusions are therefore important tools for sensory perception research to understand under what circumstances true perception breaks down^1^,^2^. In nature, animals are known to exploit the potential for creating illusions in a number of signalling or camouflage techniques^3^,^4^. However, perception of these apparent visual deceptions is poorly understood.

Colour and lightness constancy mechanisms allow animals to perceive the colour or lightness of an object as remaining constant, irrespective of varying light intensities^5^. Colour or lightness simultaneous contrast can be regarded as a breakdown in constancy mechanisms, and often causes spectrally identical targets to appear different depending on the spectral output of adjacent surrounds or the amount of illumination that they are perceived to be under.

For example, in Purves and Lotto’s lightness cube illusion^1^ (Fig. 1i), which is highly effective and intriguing for humans, we perceive the ‘orange’ tile in the shadow (A) and ‘brown’ tile on the top surface (B) as different, although they have identical spectral reflectance as shown when a mask is placed over the scene (Fig. 1ii). The ‘orange’ tile in the shadow is perceived to be brighter and a different colour because our brain considers it to be in shade and we compensate for the shadow of the cube. The brown tile on the top surface looks as if it is under bright light, so the brain assumes it is darker. The perception of the image suggests that the dark brown tile on the top is a poorly reflective surface under bright light, whereas the bright orange one at the side means a highly reflective surface in shadow. The illusion is particular striking as humans categorize orange and brown to be very different colours, rather than lighter or darker versions of one another^6^,^7^.

Such perceptual effects appear to be widespread within the animal kingdom: simultaneous colour and brightness contrast effects have been demonstrated in animals such as bees^8^, butterflies^9^,^10^, and fishes^11^,^12^, using spectrally similar targets embedded in differently coloured immediate surrounds. However, little work has been conducted to investigate how animals perceive complex illusory stimuli comprising multiple colour patches and are presented as an entire scene^1^,^13^. By studying the perceptual capabilities of animals, we hope to elucidate the fundamental principles of visual systems.

Moreover, from an ecological pespective, visually-guided animals constantly make decisions about foraging, mating, or predation using colour signals in visually heterogenous environments^14^. The colours against which objects are viewed and the illumination they are viewed under can make coloured objects look more or less similar and impact decisions made in behavioural interactions. Furthermore, some animals make use of visual illusions to facilitate their own signalling behaviour (reviewed in ref. 3). For example, male great bowerbirds (*Ptilonorhynchus nuchalis*) actively improve their mating success by creating a forced perspective illusion of the objects used in their bower court, and exhibit particular colours that activate the inducing effects of chromatic adaptation of the females’ eyes when viewing the display through the bower avenue^15^,^16^.

In this study, we examined whether coral reef fish, triggerfish (*Rhinecanthus aculeatus*; family Balistidae, also referred to as Picasso Triggerfish) perceived the shifts in coloured targets in the lightness cube illusion (Fig. 1i). We did this by training a fish to approach and tap at coloured targets, and then embedded these targets within a complex scene to determine whether their perception of a shadow would alter the way in which the coloured target was perceived.

## Experimental Procedures

We used eight triggerfish for this study, ranging from 5.3 to 15.8 cm standard length (SL). These fish are omnivorous, and inhabit shallow reef and sub-tidal reef flats throughout the Indo-Pacific region. The visual system of this species has three spectral sensitivities (λ_max_: single cone at 413 nm; double cone at 480 nm and 528 nm^17^). Fish were held in individual aquaria (45.5 × 44.5 × 80.5 cm or 41.5 × 29.5 × 79 cm) for the duration of experimental trials at the University of Queensland, St Lucia from June 2015 to January 2016. This work was approved by and performed in accordance with guidelines from the University of Queensland Animal Welfare Ethics Committee (SBS/111/14/ARC). Illumination was provided with LED lights fitted above the tanks (KR96–48 White EcoLamps Inc.) (Supplementary Figure S2).

**Figure 1:**
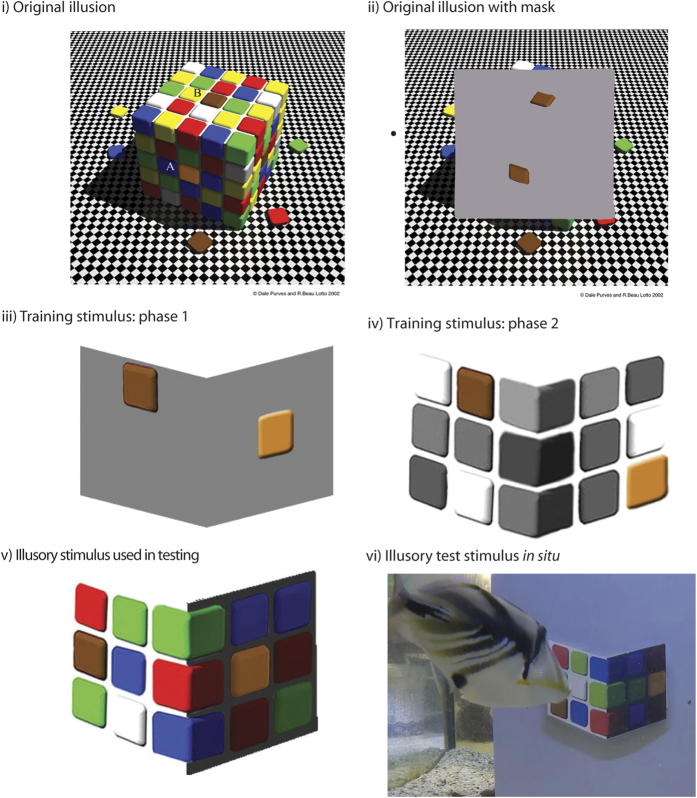
Lightness cube illusion as per^1^: (i) unmasked and (ii) masked to demonstrate that the spectral properties of the squares A and B are identical when viewed without a shadow; (iii–vi) training and testing stimulus used in the experiment.

We created visual stimuli for the experiment from the high resolution image of the illusion available from^18^. The illusion was then modified in Adobe Photoshop CS4, printed with an EPSON Artisan 1430 printer onto Kodak Premium Matte Photo paper and laminated. The visual stimuli were attached to a grey PVC feeding board with Velcro dots and positioned vertically at one end of the tank (Fig. 1vi). An opaque divider was placed in the centre of the tank whilst the stimulus board was placed into position. We first trained four fish to peck and receive a food reward (small piece of squid mantle) from an orange square target that was placed on a grey background, with a brown square as a distractor (Fig. 1iii). The other four fish were trained to receive a food reward when pecking at the brown square, with the orange square as a distractor. The position of both brown and orange squares was randomised throughout the training phase. In all training stimuli, the orange and brown target squares had differing spectral outputs (Supplementary Figure S1), and throughout the manuscript are referred to without quotation marks. In the illusory stimuli, the brown square in the shadow (Fig. 1i A) is perceived to be ‘orange’; and in this case, we use quotation marks, as the spectral output is the same as the brown target. For training, the orange square was matched to be perceptually similar to perception of the ‘orange’ tile in the illusory stimuli (Fig. 1v); however, this was from a human perspective. Fish were trained twice a day, during a morning and afternoon session, and a single session contained six consecutive training tests.

Fish were considered to have reached their training criteria when they attained >85% success rate over 8 consecutive sessions (n = 30 trials). They then progressed to training phase 2, in which the orange and brown squares were embedded in a more complex achromatic checkerboard surround (Fig. 1iv). We used a number of different training stimuli in which the position of the orange and brown squares, and the different achromatic intensities of the grey squares were randomised, to ensure that no luminance contrast cues were learnt. Again, fish were required to approach and peck their target square to receive a food reward, and once they had received a success rate of 85% over 8 consecutive sessions (n = 30 trials) they proceeded to the illusion testing. Whilst there was some variation in the time that fish took to learn the task, training was generally achieved in 4–6 weeks.

During testing, the illusion (Fig. 1v,vi) was presented to each fish between 7 and 18 times (total n = 106) and we recorded whether they pecked the brown or ‘orange’ target first (a video of this behaviour is presented in Supplementary Information). This usually occurred within a few seconds, but could take up to 2–3 min. If they pecked other coloured squares (red, green, blue, white) more than 5 times before they pecked the brown or ‘orange’ target, this session was disregarded and approximately 5 reinforcement trials were conducted to encourage correct behaviour. This occurred in 3 out of the 8 fish, on no more than 3 occasions during the experiment. The position of the brown and ‘orange’ squares were randomised, as was whether the perceived shadow side was placed on the right or left side. During testing no food was rewarded to ensure that operant conditioning of the fish was not disrupted. We therefore conducted phase 2 reinforcement training (n = 4) in between testing, and both reinforcement training and testing were conducted only once per day to promote fish accuracy and motivation for the task.

**Figure 2:**
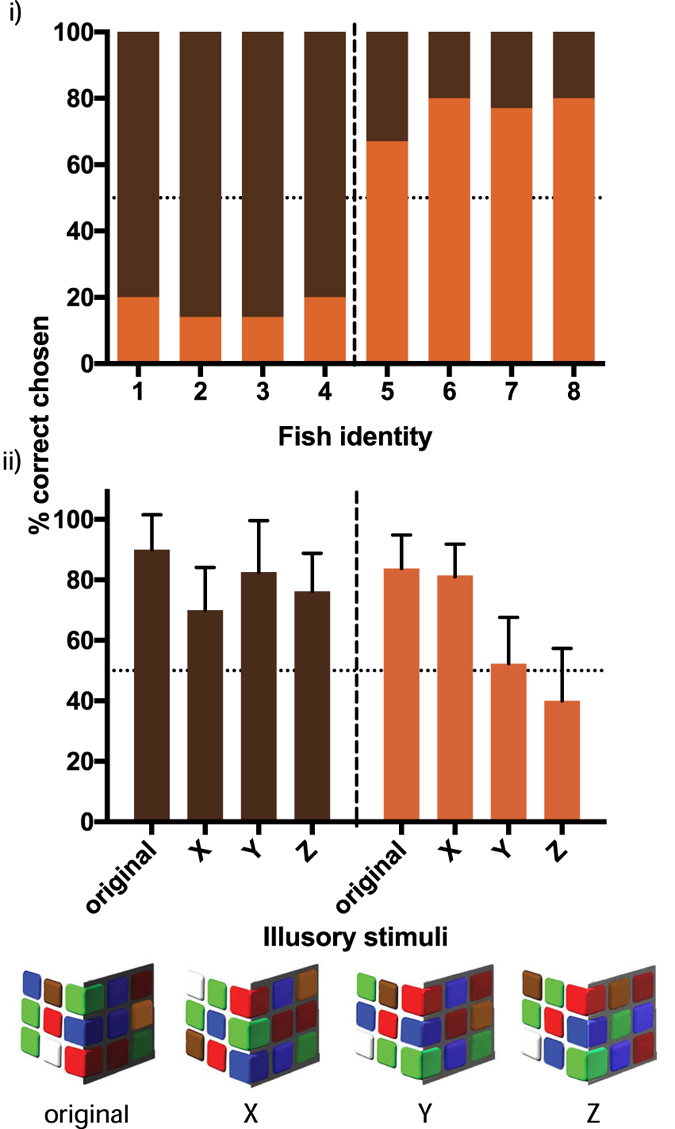
(i) Percentage (%) of targets that were selected by each fish when presented with the illusory stimuli. Brown bars indicated that the brown targets (A on Fig. 1i) were selected, orange bars indicate that illusory ‘orange’ targets (B on Fig. 1i) were selected. (ii) Percentage of ‘correct’ targets on the cube illusion stimuli with increased lightness of shadow to investigate when the ‘orange’ square becomes indistinguisable from the brown square. Brown bars indicate results from fish that were trained to brown targets; orange bars indicate fish trained to orange targets. Bars indicate mean whilst error bars represent one standard error.

Once the first experiment was conducted, we continued testing the triggerfish to determine when the illusions began to potentially breakdown for the fish, and examine when fish perceived the illusory brown and ‘orange’ to have the same spectral output. To do this, we altered the illusory stimuli so that the brightness of the original stimuli background was increased resulting in a perceptual shift in brightness of the illusory ‘orange’ target so it appeared closer to the brown target (Fig. 2iii). Individuals were tested 22–30 times (ten per stimulus) (total n = 132), and the choice of target recorded. Fish were tested once a day, with one session consisting of two to three reinforcements before a single illusion test was conducted.

Data was analysed using a generalised linear mixed model (binomial distribution) using RStudio^19^ with the package lme4^20^. The response variable was coded depending on which target was chosen (‘orange’ or brown). Fish ID was included in the model as a random factor, whilst the colour fish were trained to was a fixed factor. Fish size was included in the model as a covariate, but was non-significant (P > 0.05).

## Results

Fish that were trained to brown targets (Fish 1-4), chose the brown target on the illusion significantly more than the illusory ‘orange’ square (Z = −2.02, p = 0.04); fish that were trained to orange targets (Fish 5-8) chose the illusory ‘orange’ targets more frequently (Z = −1.59, p = 0.11) (Fig. 2i).

When the perceived shadow was made lighter on the illusory stimuli (Fig. 2iii), fish trained to brown continued to choose the brown target significantly more than the illusory ‘orange’ (Z = −2.83, p = 0.005), irrespective of shadow lightness (Z < −0.66, p > 0.51; Fig. 2ii). However, fish trained to orange, only chose the illusory ‘orange’ significantly more than the brown target when presented with the original stimulus and stimulus X. When presented on stimulus Y and Z, the amount of times they chose the illusory ‘orange’ was not significantly different from random (50% of the time) (stimulus Y: Z = 0.98, p = 0.33; stimulus Z: Z = −0.98, p = 0.31; Fig. 2ii).

## Discussion

Our study supports the hypothesis that fish experience perceptual shifts in the way in which target colour patches are viewed in a complex lightness illusion, due to the perceived amount of illumination in different regions of a scene. Fish that were trained to orange targets were more likely to choose the target stimulus viewed in the shadow of the illusory cube. We assume that this is due to perceptual mechanisms comparing the spectral output of the target object to the surrounding illumination. The fish trained to the brown targets continued to select the brown targets outside the perceived shadow. When the shadow was lightened so that the ‘orange’ target shifted back to its ‘true’ spectral output and was perceived to be brown, the fish began selecting the brown and orange equally. This has important implications for animals that view objects within and out of shadows, as this may impact behavioural decisions for many animals. Interestingly, Picasso triggerfish and many other triggerfish have orange colour patches on their body (Supplementary Figure S3); therefore, how they perceive conspecifics and competitors may be impacted by shadows in their environment.

How and why colour contrast and constancy mechanisms occur in animals has long fascinated vision scientists, and lightness perception is of particular interest when considering how animals process visual information^21^. There are many theories of how animals perceive illusions, including the *retinex* model first proposed by Land^22^,^23^ that suggest interactions occur laterally among neurons in the retina and as such, the viewed scene is broken down into different spectral regions, which are then compared relative to one another before signals are sent to the visual cortex. While ideas surrounding the mechanisms have changed, the central tenet of the retinex model remains. Higher stages of neuronal processing in the visual cortex have large receptive fields capable of summing predominant spectral returns from areas of the retina, which correspond to different areas of a scene (for review see ref. 24). The wholly empirical approach to vision proposes that perceived differences are based on empirical information (e.g. past experience) of not just the target stimulus alone, but critically including the context in which it is viewed^25^. In the last few years, this framework has received much attention^25^,^26^. Corney and Lotto^5^ suggested that animals using visually-guided behavior should also be susceptible to illusions as they experience the same signal variation in their visual environment.

Whether contrast mechanisms occur via processes in the retina, in the visual cortex, or based on past experience, it is certainly interesting to demonstrate that non-human species also perceive illusive objects in apparently the same way as we do as humans. If their neural processing is less complex, this perhaps argues for the idea that at least part of the fundamental mechanisms for illusory perception happens early on in the visual pathway. Indeed, the ability to detect lightness illusions has now been shown in butterflies^10^, guppies^12^ and now in triggerfish.

## Additional Information

**How to cite this article**: Simpson, E. E. *et al.* Coral reef fish perceive lightness illusions. *Sci. Rep.* **6**, 35335; doi: 10.1038/srep35335 (2016).

## Supplementary Material

Supplementary Information

Supplementary Video
