# Supplementary material for: Coral reef fish perceive lightness illusions
Source: Sci Rep. 2016 Oct 17;6:35335. doi: 10.1038/srep35335 (PMC5066220; doi:10.1038/srep35335)

<u>Supplementary Information</u>

Fooling 'Picasso': coral reef fish perceive lightness illusions

Elisha E. Simpson[1], N. Justin Marshall[2], Karen L. Cheney[1,2*]

[1]School of Biological Sciences, The University of Queensland, Brisbane, Queensland, 4072, Australia
[2]Queensland Brain Institute, The University of Queensland, Brisbane, Queensland, 4072, Australia

Figure S1: Spectral reflectance curvues of colours used in illusionary stimuli

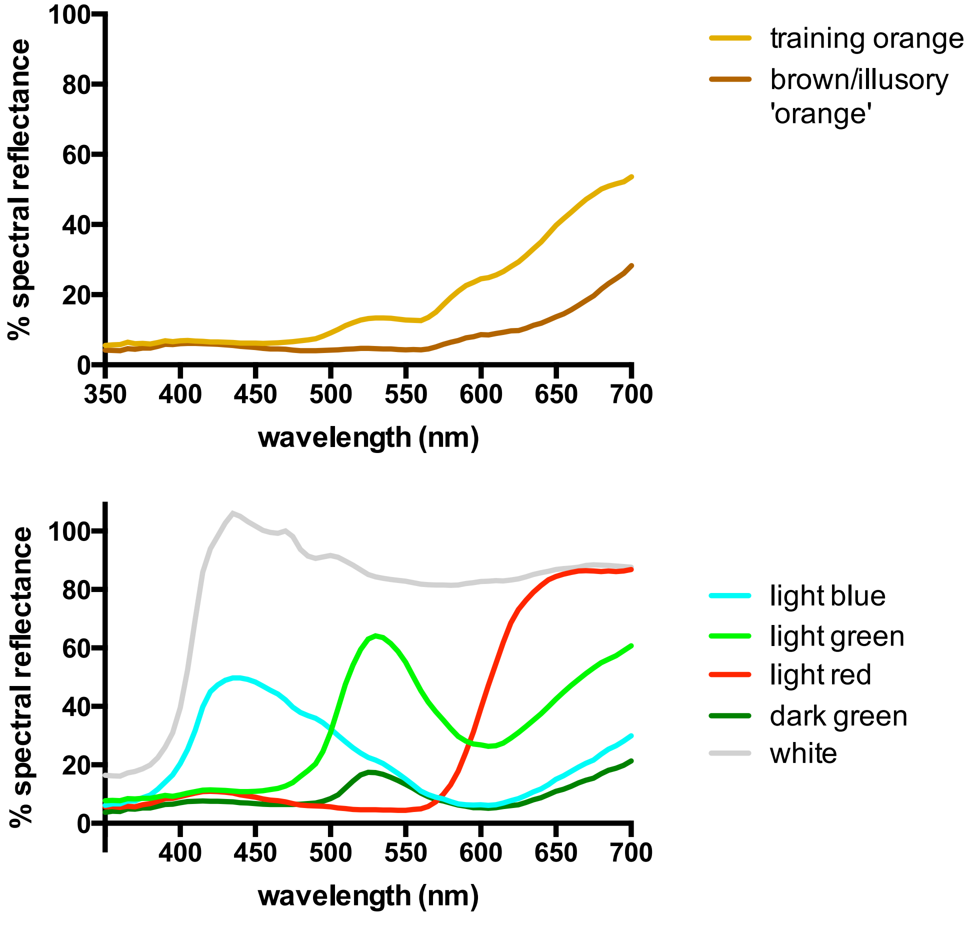

Figure S2: Spectral irradiance measurements of LED lights used in Experiment.

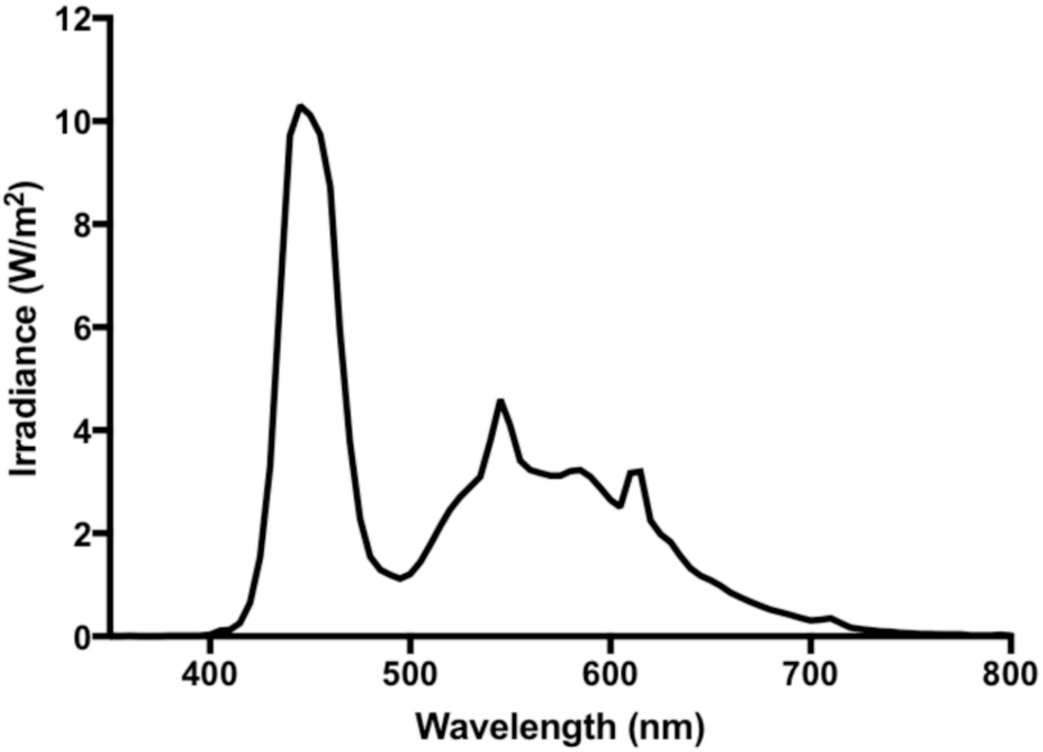

Figure S3*:* Colour patterns of i) Picasso triggerfish *Rhinecanthus aculeatus*, and ii) Titan triggerfish *Balistoides viridescens* and iii) Orange-lined triggerfish *Balistapus undulatus* (photograph by Leonard Low). Photographers of triggerfish from top to bottom: Adrian Pingstone (https://commons.wikimedia.org/wiki/File:Picasso.triggerfish.arp.jpg), Jan Derk (https://commons.wikimedia.org/wiki/File:Titan_Triggerfish.jpg), Leonard Low (https://commons.wikimedia.org/wiki/File:Balistapus_undulatus.jpg). Images i) and ii) were downloaded from the public domain; image iii) is licensed for reuse under the Creative Commons Attribution-Share Alike 2.0 Generic License. To view a copy of this license, visit http://creativecommons.org/licenses/by-sa/2.0/

i)

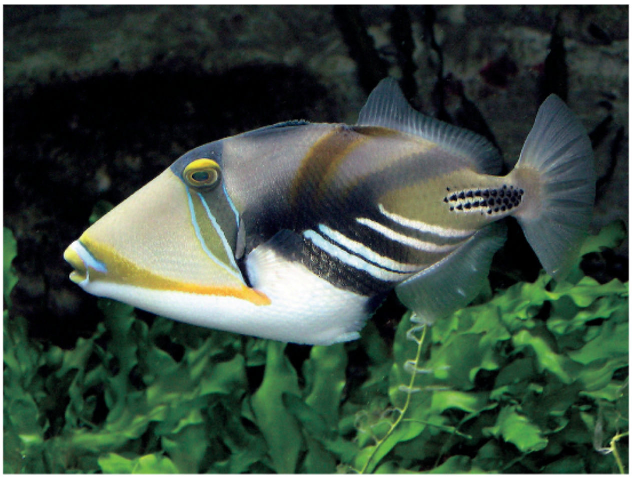

ii)

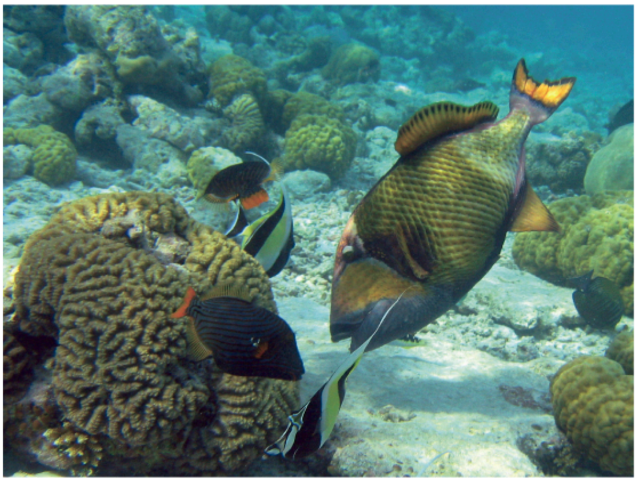

iii)

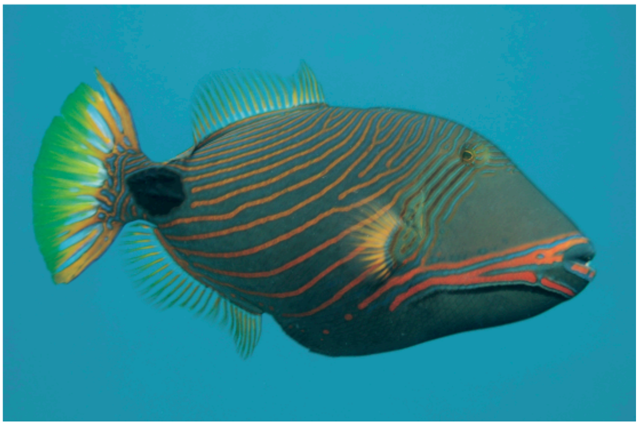

Supplement: Supplementary Information [file srep35335-s1.pdf]
